# Electrolyte Design for Low-Temperature Li-Metal Batteries: Challenges and Prospects

**DOI:** 10.1007/s40820-023-01245-9

**Published:** 2023-11-29

**Authors:** Siyu Sun, Kehan Wang, Zhanglian Hong, Mingjia Zhi, Kai Zhang, Jijian Xu

**Affiliations:** 1 Department of Chemistry, City University of Hong Kong, Hong Kong, 999077 People’s Republic of China; 2 State Key Laboratory of Silicon Materials, School of Materials Science and Engineering, Zhejiang University, Hangzhou, 310027 People’s Republic of China; 3 State Key Laboratory of Advanced Chemical Power Sources, Key Laboratory of Advanced Energy Materials Chemistry (Ministry of Education), Haihe Laboratory of Sustainable Chemical Transformations, Collaborative Innovation Center of Chemical Science and Engineering (Tianjin), College of Chemistry, Nankai University, Tianjin, 300071 People’s Republic of China; 4 Department of Chemical and Biomolecular Engineering, University of Maryland College Park, College Park, MD 20742 USA

**Keywords:** Solid electrolyte interphase, Li metal, Low temperature, Electrolyte design, Batteries

## Abstract

Electrolyte design holds the greatest opportunity for the development of batteries that are capable of sub-zero temperature operation. To get the most energy storage out of the battery at low temperatures, improvements in electrolyte chemistry need to be coupled with optimized electrode materials and tailored electrolyte/electrode interphases. Herein, this review critically outlines electrolytes’ limiting factors, including reduced ionic conductivity, large de-solvation energy, sluggish charge transfer, and slow Li-ion transportation across the electrolyte/electrode interphases, which affect the low-temperature performance of Li-metal batteries. Detailed theoretical derivations that explain the explicit influence of temperature on battery performance are presented to deepen understanding. Emerging improvement strategies from the aspects of electrolyte design and electrolyte/electrode interphase engineering are summarized and rigorously compared. Perspectives on future research are proposed to guide the ongoing exploration for better low-temperature Li-metal batteries.

## Highlights

- A critical assessment of electrolytes’ limiting factors, which affect the low-temperature performance of Li-metal batteries.
- Summary of emerging strategies to improve low-temperature performance from the aspects of electrolyte design and electrolyte/electrode interphase engineering.
- Perspectives and challenges on how to develop creative solutions in electrolytes and correlative materials for low-temperature operation.

## Graphical abstract

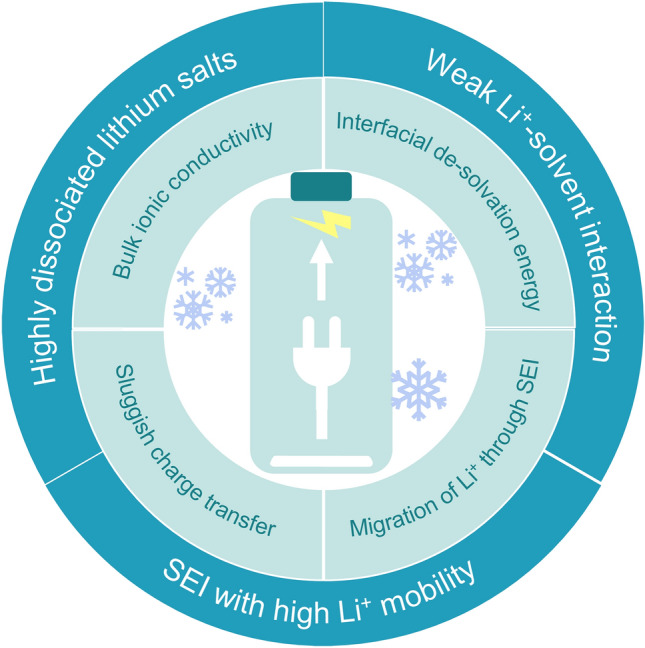

## Introduction and Scope

Under the burgeoning call for a green energy transition, batteries are being used in an ever-increasing variety of applications ranging from electric vehicles to large-scale energy storage. However, commercial lithium-ion batteries (LIBs) suffer from significant capacity loss, poor cycle life, difficulty in charging, and even cell failure when operated at low temperatures (i.e., below − 20 °C). For potential applications in satellites, space probes, and submarine missions—all requiring reliable battery performance in extremely cold environments—the stable operation of LIBs at low temperatures is of paramount importance. The slow Li-ion diffusion within the graphite anode is believed to be one of the main reasons for the poor low-temperature performance of LIBs [1]. From this perspective, Li-metal anode with a plating/stripping process provides benefits over graphite anode with Li-ion intercalation/deintercalation mechanism for low-temperature applications [2]. Moreover, Li metal anode has a high theoretical capacity (3860 mAh g^−1^) [3], far exceeding the capacity of graphite (372 mAh g^−1^) [4].

The operation of Li-metal batteries (LMBs) at low temperatures is still confronted with a series of problems associated with not only bulk electrolytes but also electrolyte-derived electrolyte/electrode interphases [5–8]. All these factors result in significant performance deterioration of LMBs at low temperatures, hindering their adoption in cold regions. Although external heating can be used to increase the battery’s real operating temperature, it consumes extra energy and increases additional [9]. In sharp contrast, rational electrolyte design offers the most cost-effective and drop-in solution for current battery chemistries to boost the low-temperature performance of LMBs. Extensive efforts have been devoted to improvements in electrolytes by introducing low-melting point and/or low-viscosity solvents, employing highly dissociated lithium salt, and adding functional additives. It is also well-acknowledged that the electrolyte-derived solid electrolyte interphase (SEI) and cathode electrolyte interphase (CEI) play an essential role in determining low-temperature performance. There have been several reviews summarizing the knowledge about low-temperature LIBs [10, 11]. Nevertheless, few reviews on low-temperature LMBs have focused on the rational electrolyte design [12] and the electrolyte-derived electrolyte/electrode interphases as far as we know.

In this review, we first analyze the basic Li-ion transportation processes on the Li-metal anode side and then identify why LMBs’ properties degrade at low temperatures. Detailed theoretical derivations are presented, explaining the explicit influence of temperature on battery performance. Various strategies of electrolyte design are classified into three categories: i) employing highly dissociated lithium salt to enhance bulk ionic conductivity and prevent salt precipitation at low temperatures; ii) using weakly solvating solvents to minimize the de-solvation energy; iii) constructing robust SEI with high Li^+^ mobility. Finally, perspectives and challenges on future research are proposed, aiming to facilitate the cutting-edge perception for better low-temperature LMBs.

## Limiting Factors on Low-Temperature Performance of LMBs

**Fig. 1:**
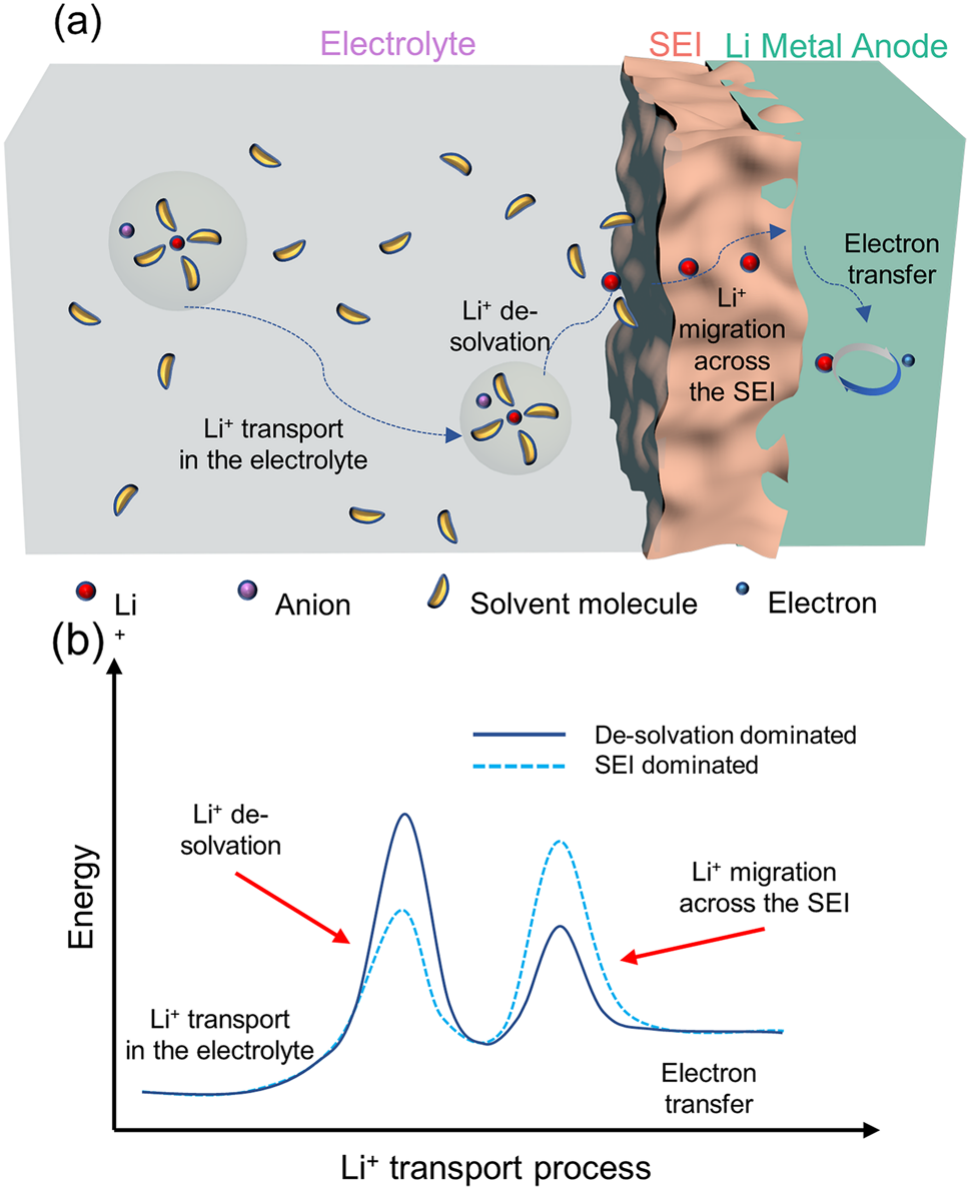
**a** Schematic diagram of Li^+^ transportation during the charging process of LMBs on the anode side. **b** The energy barrier diagram corresponds to different Li^+^ transportation steps

In the purpose of solving the performance degradation at low temperatures of LMBs, it is essential to take a deep dive into the basic Li-ion transportation steps during the charge/discharge process. Taking the challenging charging process as an example, the Li^+^ transportation on the Li-metal anode side is mainly divided into the following steps (Fig. 1a) [13]:

- Solvated Li^+^ transports in the bulk electrolyte.
- Li^+^ de-solvation at the surface of the SEI.
- The exposed Li^+^ migrates into the anode through the SEI.
- Electrode reaction occurs, and Li^+^ is reduced into Li^0^.

The energy barriers of these four steps are illustrated in Fig. 1b. At low temperatures, the rate-limiting or performance-limiting steps change dynamically at different conditions, which are discussed in detail in the following sections (Table 1).

**Table 1:** Properties of Lithium salts used in LMBs electrolyte systems

Salt	Structure	Price ($ g^−1^)	*T*_m_ (°C)	Dissociation energy (kJ mol^−1^)
LiPF_6_		~ 10	200	439
LiPO_2_F_2_		~ 10	360	/
LiFSI		> 15	124–128	344
LiTFSI		~ 5	234–238	514
LiBF_4_		~ 24	293–300	596
LiDFOB		> 13	265–271	494
LiTFPFB		/	/	252

### Bulk Ionic Conductivity

Ionic conductivity (σ) describes the ability of ion transport in the electrolyte, expressed by Eq. (1) in an ideal dilute solution [14].

1 $$\sigma \, = \, \Sigma n_{{\text{i}}} \mu_{{\text{i}}} Z_{{\text{i}}} e$$

where *n*_i_ represents free ion number, *Z*_i_ represents the valence of the ionic species, e represents the unit charge of electrons, and μ_i_ represents ionic mobility. In Eq. (1), the *Z*_i_ and *e* are constants. Therefore, *σ* is mainly affected by *n*_i_ and *μ*_i_. *n*_i_ reflects the number of ions involved in conduction, which is temperature-dependent. Note that none of the electrolytes used in real cells is ideal, extra attention is needed when applying this equation to practical electrolytes [15]. Ionic mobility *μ*_i_ can be given by Eq. (2):

2 $$\mu_{{\text{i}}} = { 1}/{6}\pi \eta r_{{\text{i}}}$$

in which* r*_i_ represents the solvation radius of ionic *i*, and *η* represents the viscosity of the solvent. According to the Vogel–Fulcher–Tammann (VFT) equation (Eq. (3)) *η* can be described as follows [16]:

3 $$\eta \, = \, \eta_{0} \exp \, \left[ {B/\left( {T - T_{0} } \right)} \right]$$

where *η*_0_, *B,* and *T*_0_ are empirical constants. As the temperature drops, the viscosity of the solvent will increase. As a result, ionic mobility will decrease, leading to a decline in conductivity directly. A typical example is that aqueous electrolytes generally freeze below zero [17, 18]. Organic electrolytes are no exception, as they exhibit poor kinetics of Li^+^ transport at low temperatures due to electrolyte freezing. In general, the conductivity reduces with decreasing temperature [19]. To mitigate the decrease in ionic conductivity caused by low temperatures, the adoption of highly dissociated lithium salts can be considered.

### Interfacial De-Solvation Energy

**Fig. 2:**
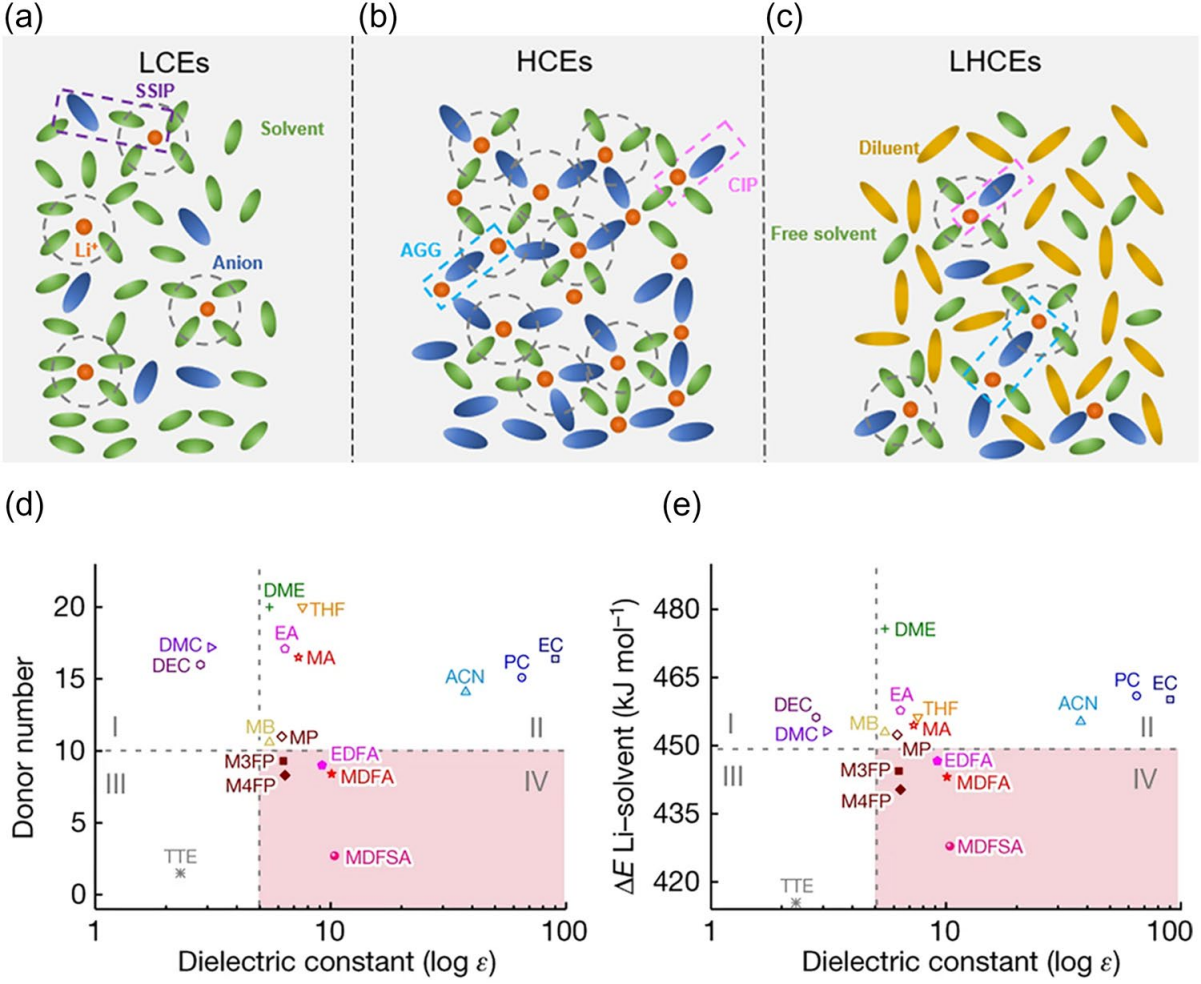
**a**–**c** Schematic Li^+^ solvation structure of **a** LCEs, **b** HCEs, and **c** LHCEs. Reproduced with permission from Ref. [23]. Copyright 2021, American Chemical Society. **d** The diagram of donor number versus dielectric constant. Reproduced with permission from Ref. [20]. **e** The calculated Li^+^-solvent binding energy versus dielectric constant. Reproduced with permission from Ref. [25]. Copyright 2023, Springer Nature

In addition to the reduced ionic conductivity, large de-solvation energy is considered to be the main factor limiting ion kinetic transport at low temperatures [20–22]. The de-solvation process, as illustrated by step (b) in Fig. 1, corresponds that the solvated Li^+^ must be de-solvated to ensure the smooth passage of bare Li^+^ through the SEI. Li ions possess different solvation structures in different electrolytes and therefore different de-solvation energies. Various electrolytes can be roughly classified into three types in terms of concentration, as shown in Fig. 2. In low-concentration electrolytes (LCEs), there is almost no anion in the solvated structure of Li^+^ as dominated by solvent separated ion pair (SSIP) (Fig. 2a). Because of the high salt/solvent ratio of high-concentration electrolytes (HCEs), anions participate in the solvated structure as coordinated contact ion pairs (CIPs) and ionic aggregates (AGGs) structures (Fig. 2b). Localized highly concentrated electrolytes (LHCEs) retain the anion-participated solvation structure without increasing the salt concentration by introducing non-solvating diluents (Fig. 2c) [23].

The de-solvation ability depends on the Li^+^-solvent binding energy and coordination number of the solvent molecules. High binding energies between Li^+^ and solvent molecules mean that the de-solvation process is difficult to carry out [23, 24]. At ultra-low temperatures, it is more difficult to cross this energy barrier. A key factor describing the Li^+^-solvent binding energy is the donor number of a solvent molecule. Semi-empirically, a solvent with a larger donor number indicates a larger Li^+^-solvent binding energy, as shown in Fig. 2d, e. The Li^+^-solvent binding energy from the DFT calculation agrees well with the donor number, suggesting donor number is a valuable parameter for preliminary evaluating the Li^+^-solvent binding energy [25]. It is also suggested that a small Li^+^ coordination number of solvent molecules corresponds to a small de-solvation energy [26]. Unfortunately, this assumption becomes more complicated when the temperature is reduced to low temperatures. Sheng et al. adopted a Density-Functional Tight-Binding based Molecular Dynamic (DFTB-MD) for calculating the number of solvated solvents in the solvated structure of Li^+^ at different temperatures [27], and the calculations showed that the coordination number of Li^+^ is temperature-dependent. For the same electrolyte, the increased coordination number at low temperatures indicates a larger de-solvation energy. One approach to reduce the interfacial de-solvation energy is to employ solvents with weak interaction with Li^+^.

### Migration of Lithium Ions Through SEI

There is a consensus that the formation of a robust SEI is the key to enabling a stable Li metal anode. But the fundamental question of the exact transport mechanism of Li^+^ across the SEI is still not fully answered due to the limitation of available characterization tools. Two possible Li^+^ transport mechanisms are proposed: one is by Fick’s law, in which Li^+^ moves within the interstitial site in the SEI mainly in the form of point defects [28]. According to Fick’s law, the diffusion coefficient reduces when the temperature decreases. It is easy to conclude that the number of times the Li^+^ crosses the barrier per unit of time, i.e., the probability of the ion moving from one gap position to another per unit of time, decreases considerably with decreasing temperature.

Another mechanism suggested that Li^+^ transport (regardless of the point defect form) relies on thermal motion to continuously migrate from one location to another. The slow transport of Li^+^ at low temperatures leads to another type of electrochemical polarization, concentration difference polarization. Like electrode polarization, concentration polarization will lead to overpotential, affecting the low-temperature performance of the battery, and may have huge consequences in severe cases. Also note that SEI is inherently anisotropic, and the slow migration of Li^+^ will exacerbate their uneven distribution in the SEI layer and thus reduce the cycling performance of the battery.

**Fig. 3:**
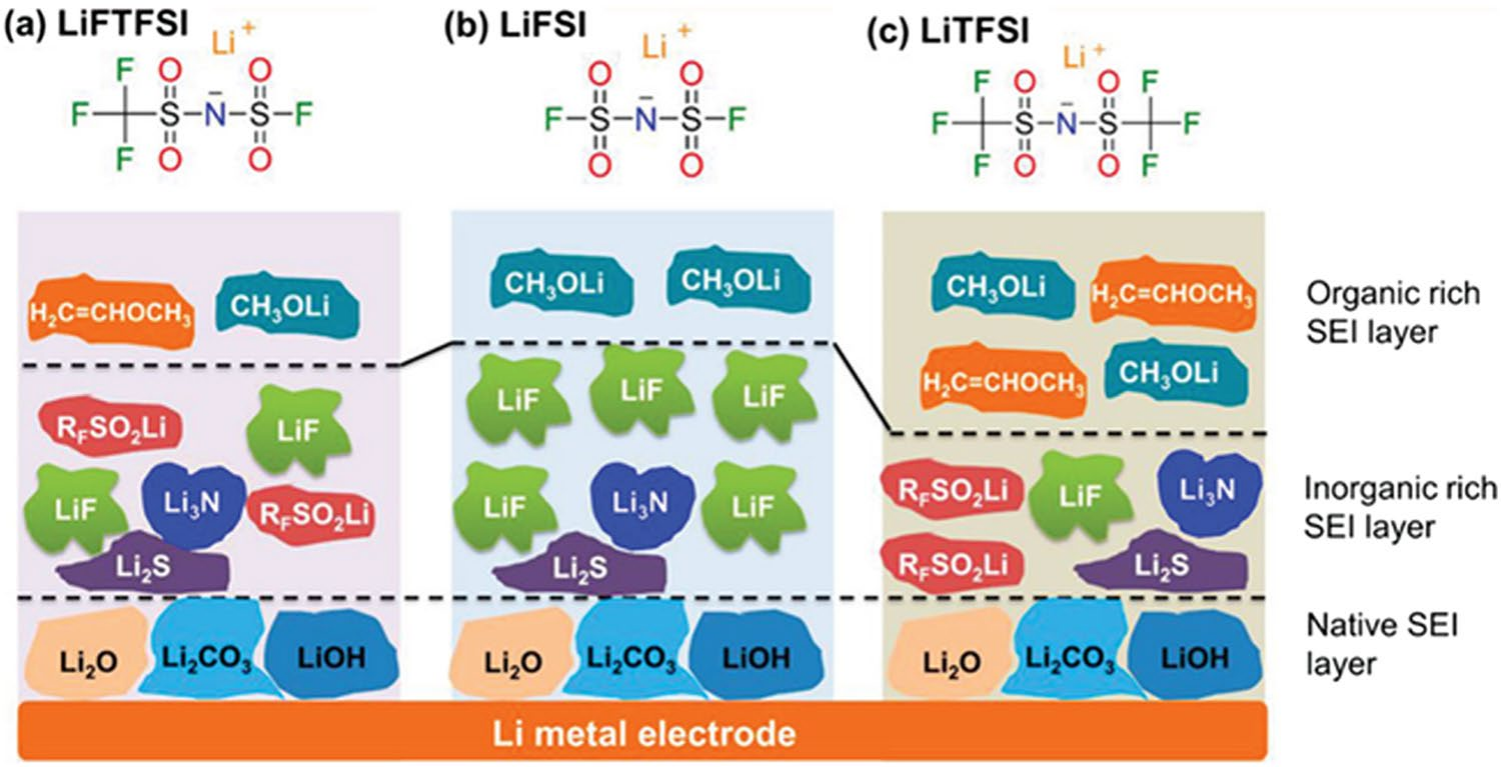
Illustration of SEI layers formed on lithium metal surfaces by three different lithium salts. **a** Lithium (fluorosulfonyl)(trifluoromethanesulfonyl)imide, LiFTFSI, **b** Lithium bis(fluorosulfonyl)imide, LiFSI, and **c** Lithium bis(trifluoromethanesulfonyl)imide, LiTFSI. Reproduced with permission from Ref. [28]. Copyright 2021, Wiley–VCH

There is no doubt that the migration of Li^+^ across SEI depends on the SEI composition. Unfortunately, SEI remains the least understood component because of the complicated SEI compositions and dynamic evolution (Fig. 3). There are ongoing controversies about the composition and structure of SEI [29]. Most recently, the SEI film was revealed to consist of a dense inorganic inner layer and an organic outer layer that is permeated by the electrolyte via cryo-electron microscopy and spectroscopy [30]. Several reviews and progress reports have been devoted to summarizing the SEI compositions and structures [31–33]. An accelerated transport of Li^+^ can be achieved by increasing the proportion of inorganic components SEI.

### Sluggish Charge Transfer

**Fig. 4:**
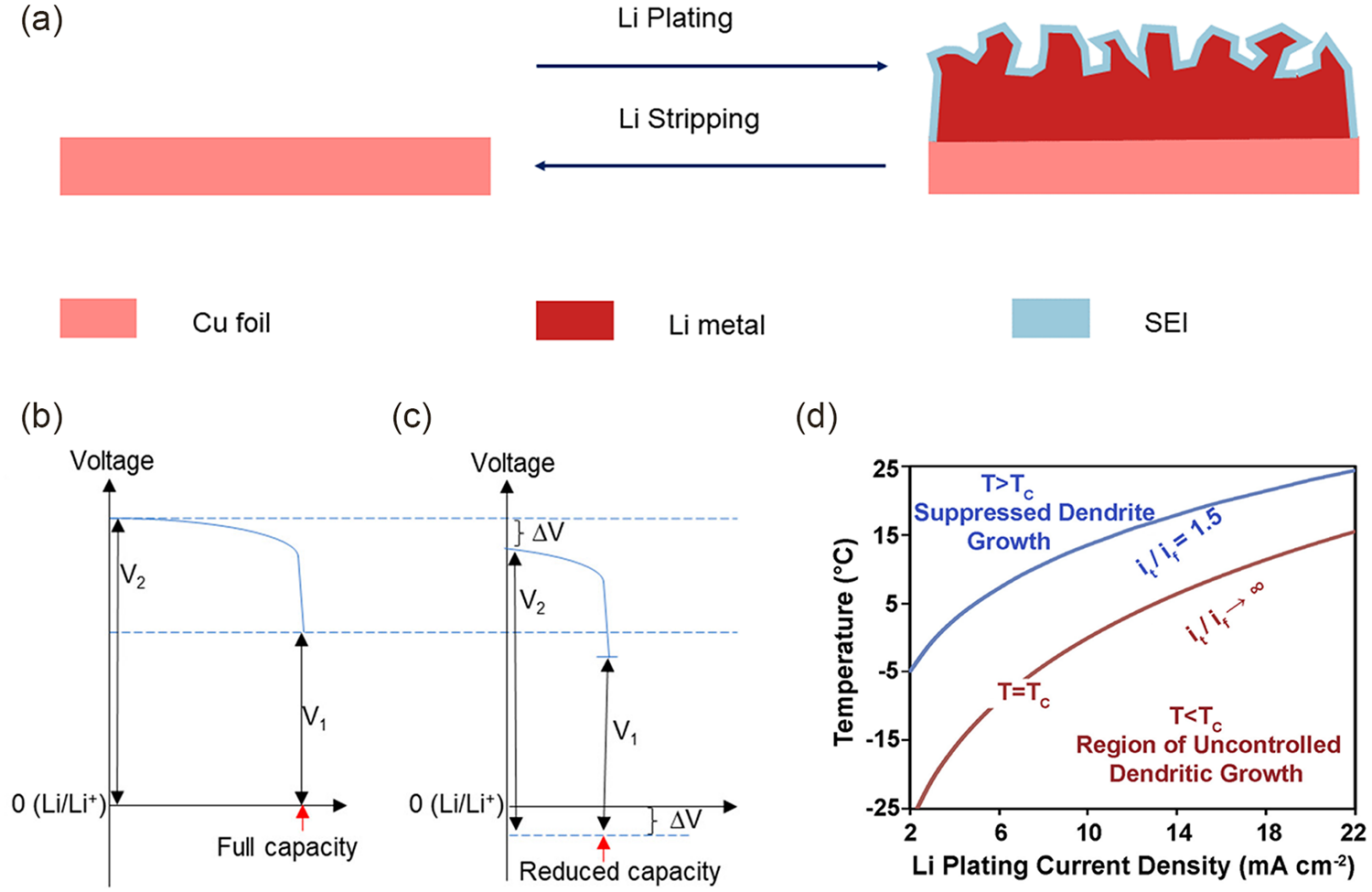
**a** Electroplating/stripping process of Li metal (golden). Voltage profiles of LMBs without **b** and with **c** a high impedance passivation layer on Li metal anode. Reproduced with permission from Ref. [40]. Copyright 2020, American Chemical Society. **d** Variation of the dendrite growth rate ratio i_t_/i_f_ with temperature and Li plating current density. Reproduced with permission from Ref. [29]. Copyright 2020, Elsevier

After reaching the surface of the electrode, the Li^+^ receives an electron provided by the external circuit, and charge transfer occurs [34]. LMBs alternate the plating/stripping process during the charge/discharge cycle (Fig. 4a) [35]. Electron transfer occurs at the interface between electrolyte and electrode as shown in Eq. (4):

4 $${\text{Li}}^{ + } + {\text{e}}^{ - } \leftrightarrow {\text{Li}}^{0}$$

Thermodynamically, the rate of all chemical reactions is closely related to temperature. The charge transfer becomes slow at low temperatures, which can be expressed by the Arrhenius formula (Eq. 5) [36]:

5 $$k = \, A \, \exp \, \left( {E_{{\text{a}}} /{\text{RT}}} \right)$$

where *k* is the rate constant of the chemical reaction, and *E*_a_ is the activation energy of electron transfer. The reaction rate becomes slow with the decrease of temperature, which leads to electrode polarization. The exchange current density is a measure that describes the kinetic properties of the cells. The greater the polarization of the electrodes, the smaller the exchange current density, which means that the battery kinetics are sluggish. In addition, polarization will cause the battery to be undercharged, prolonging the charging time. Severe polarization will also increase the overpotential, causing severe cell heating and Li dendrites [37, 38]. Li dendrites can bring many adverse effects to the LMBs, such as reducing Coulombic efficiency (CE) and shortening cell lifespan [39]. As illustrated in Fig. 4b, c, the ohmic potential drop on the Li metal anode containing a high impedance passivation layer causes a drop in the positive and negative voltages versus Li/Li^+^ [40]. It was proposed that the ratio of the dendritic tip current density (*i*_t_) to the flat substrate surface current density (*i*_f_) quantifies the dendritic growth rate. When the temperature is higher than the critical growth temperature T_c_ (at the rate *i*_t_/*i*_f_ → ∞), the dendritic growth is inhibited (Fig. 4d) [29]. Furthermore, the presence of a large overpotential means that the cells require more energy than thermodynamically expected to drive the reaction. Therefore, the generation of such electrode polarization must be suppressed to ensure the safety of LMBs at low temperatures.

The dependence of the above four factors on temperature cannot be ignored, and they are not relatively independent. Together, they lead to poor performance of LMBs at low temperatures, which brings difficulties to applying LMBs at low temperatures.

**Fig. 5:**
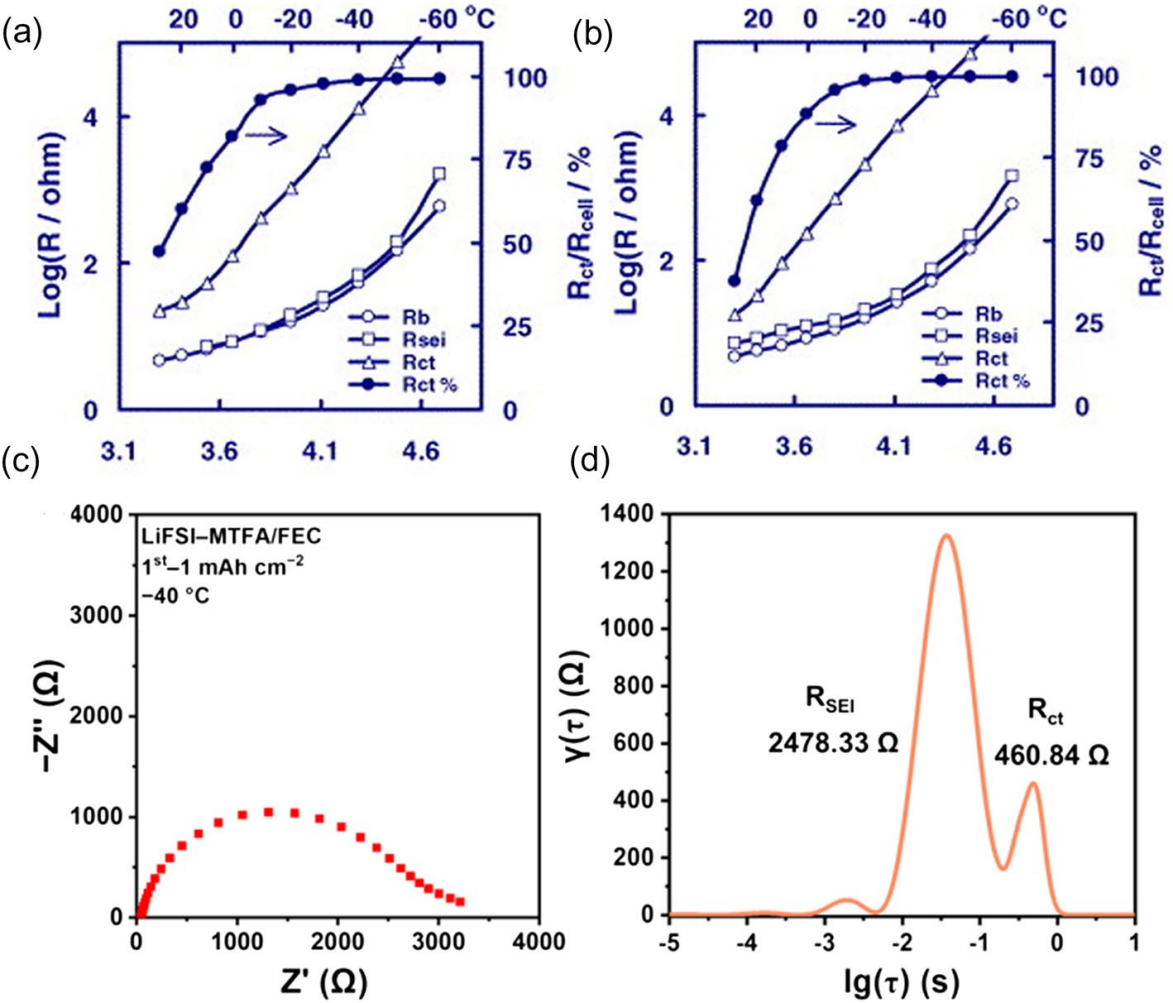
Temperature dependence of the *R*_b_, *R*_SEI_, *R*_ct_ as well as the R_ct_ percentage. **a** 3.87 V and **b** 3.45 V. Reproduced with permission from Ref. [43]. Copyright 2004, Elsevier. **c** EIS of the Li||Cu cells with LiFSI–MTFA/FEC electrolyte at − 40 °C after initial deposition (0.5 mA cm^−2^, 1.0 mAh cm^−2^) and **d** corresponding DRT analysis. Reproduced with permission from Ref. [45]. Copyright 2023, Springer Nature

Which of these four processes is the rate-limiting step at low temperatures? Initially, it was widely believed that the de-solvation process was the rate-limiting step [41–43]. However, recent research has introduced a different perspective, suggesting that Li^+^ diffusion within the SEI may be the limiting step [44, 45]. Xu et al. [43] conducted impedance calculations on a graphitic anode after cycling using an ethylene carbonate (EC) and ethyl methyl carbonate (EMC) mixture-based electrolyte. As shown in Fig. 5a, b, the SEI resistance (*R*_SEI_) is found to be smaller than the charge transfer resistance (*R*_ct_) at room temperature. Furthermore, as the temperature decreases, the ratio of *R*_ct_ to the total impedance increases. It is worth mentioning that carbonate-based electrolytes are known to form a stable SEI with graphite, establishing an important context for these findings. On the other hand, Weng et al. [45] demonstrated in their study, depicted in Fig. 5c, d, that the *R*_ct_ is considerably smaller than the *R*_SEI_ at low temperatures. The discrepancy becomes more pronounced as the temperature decreases. However, it is crucial to note that their study employed a fluoroethylene carbonate (FEC)-based electrolyte, which tends to decompose and generate organic components after cycling in LMBs, leading to the formation of a larger *R*_SEI_. This could be the reason for their differing viewpoints and this disparity highlights why most of the electrolytes discussed in this study are ether-based rather than carbonates-based for LMBs. Therefore, it is crucial to form an SEI rich in inorganic components by adjusting the electrolyte formulation.

## Strategies to Improve Low-Temperature Performance of LMBs

Generally, the electrolyte for commercial LIBs is ~ 1 M lithium hexafluorophosphate (LiPF_6_) in a mixture of ethylene carbonate (EC) and linear carbonate solvents. However, these carbonate-based electrolytes are not suitable for LMBs because of their poor stability toward Li metal anode with high reactivity [46]. Choosing a new electrolyte that is suitable for LMBs is essential. At its most fundamental level, the electrolyte should consist of solvents with low freezing points. When the temperature approaches or falls below the freezing point of the electrolyte, the fluidity of the electrolyte becomes restricted, thereby impacting the battery’s performance. Extremely low temperatures can cause the electrolyte to solidify, hindering the transport of lithium ions within the battery, thus reducing both discharge capacity and charging rate. A great deal of research has been done to explore electrolytes that can provide excellent performance for LMBs and maintain it at low temperatures.

### Highly Dissociated Lithium Salts

**Fig. 6:**
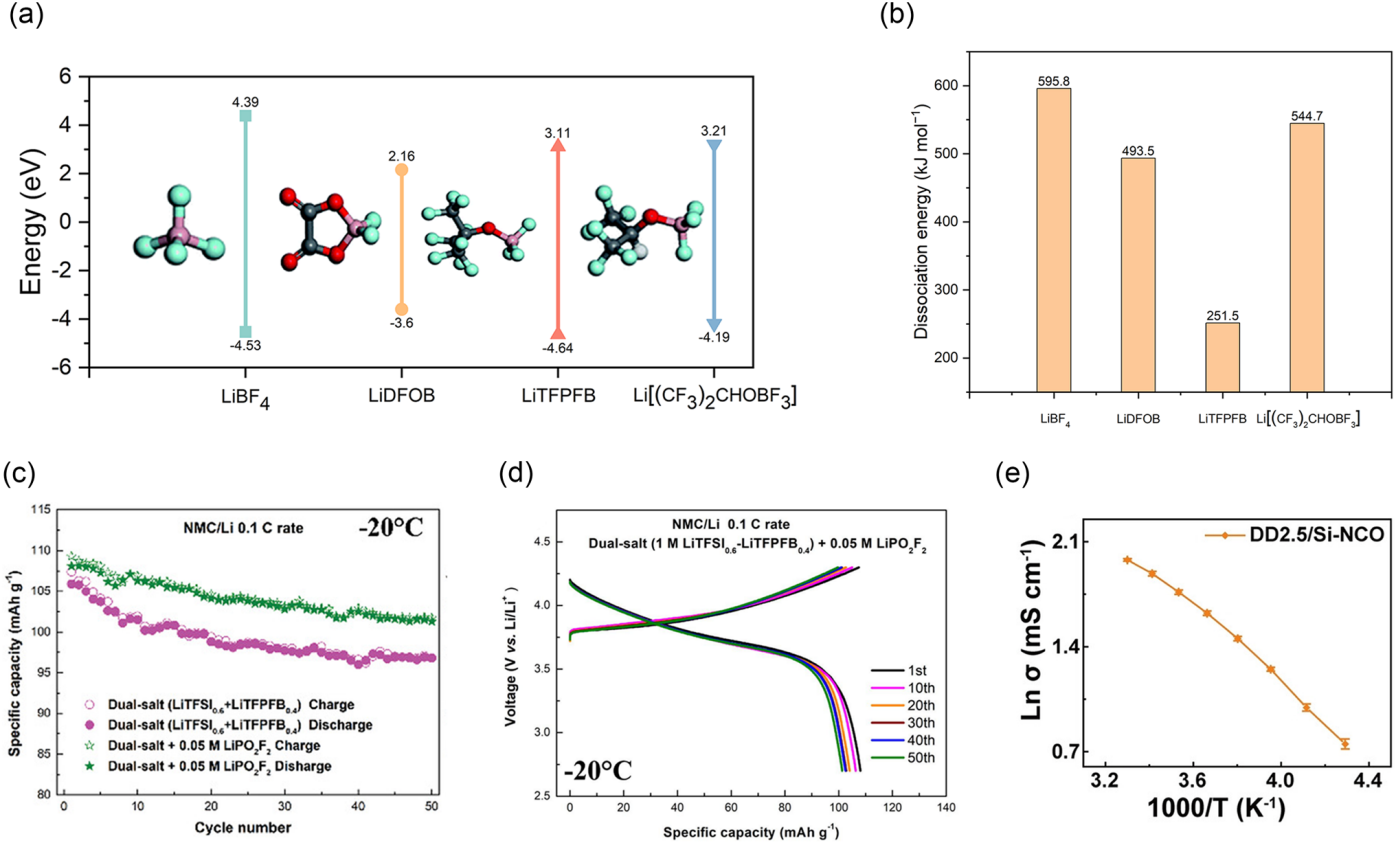
**a** HOMO and LUMO energies of LiBF_4_, LiDFOB, LiTFPFB, and Li[(CF_3_)_2_CHOBF_3_]. **b** Dissociation energies of LiBF_4_, LiDFOB, LiTFPFB and Li[(CF_3_)_2_CHOBF_3_] [49]. **c** Cycling performance of Li||NMC532 cells using the dual-salt electrolyte at − 20 °C with and without 0.05 M LiPO_2_F_2_ additive at a rate of 0.1 C, respectively. **d** The representative charge/discharge curves of Li||NMC532 cells (2.7–4.3 V) at different cycles using 1 M LiTFSI_0.6_–LiTFPFB_0.4_ dual-salt electrolyte with 0.05 M LiPO_2_F_2_ additive, respectively, at − 20 °C at 0.1 C rate. Reproduced with permission from Ref. [54]. Copyright 2019, Wiley–VCH. **e** The variation of ionic conductivity in DOL/DME mixed electrolyte as a function of temperature. Reproduced with permission from Ref. [58]. Copyright 2023, Wiley–VCH

Highly dissociated lithium salts have been extensively studied to enhance conductivity and improve battery performance. This is attributed to the presence of electron-withdrawing functional groups, which facilitate the distribution of negative charges, thereby reducing the lattice energy of the salt and promoting ion dissociation. Using highly dissociated lithium salts at low temperatures can enhance the electrolyte’s conductivity and alleviate the slow Li^+^ transport. For example, the -CF_3_SO_2_ group in the structure of lithium bis (trifluoromethane sulfonyl) imide (LiTFSI), has a strong electron-absorbing effect, which intensifies the departure of negative charges from the domain and reduces ion association pairing, resulting in a high degree of dissociation [47]. The electrolyte of 7 M LiTFSI per solvent (1,3-dioxolane (DOL): dimethoxyethane (DME) mixed 1:1 by volume) exhibited a conductivity of approximately 11.1 mS cm^−1^ at − 20 °C and it showed great rate performance in the temperature range of − 20 to 0 °C [48]. Qiao et al. [49] calculated the dissociation energies of LiBF_4_, lithium difluoro(oxalate)borate (LiDFOB), Li[(CF_3_)_3_COBF_3_] (LiTFPFB), and Li[(CF_3_)_2_CHOBF_3_] (Fig. 6a, b) and found that LiTFPFB has the lowest dissociation energy.

The dual salt system is an electrolyte system that uses two lithium salts that interact synergistically to impact battery performance. Cheng et al. [50] investigated the surface reaction mechanisms of the LiDFOB + LiTFSI/DME dual salt electrolyte system on LMB. They found that, on the one hand, the rapid decomposition of LiTFSI can protect LiDFOB while consuming a certain amount of free Li^0^, preventing the generated free boron atoms from coordinating with Li^0^ and losing reactivity. On the other hand, the decomposition of LiDFOB generates boron atoms that can trigger the decomposition of DME and polymer growth. Shangguan et al. [50] proposed a dual salt electrolyte formulation. They synthesized LiTFPFB and combined it with LiTFSI by adding lithium difluorophosphate (LiPO_2_F_2_) to this dual-salt electrolyte. LiTFPFB containing a large volume of anions not only improved the conductivity of the electrolyte but also inhibited the corrosion of the Al current collector induced by LiTFSI. The Li||LiNi_0.5_Mn_0.3_Co_0.2_O_2_(NMC532) cells using this electrolyte showed excellent performance after charging and discharging at − 20 °C. After 50 cycles at 0.1 C rate, the discharge capacity slightly changed from 108.1 to 101.3 mAh g^–1^ and has an ultra-high average CE of 99.7%, as shown in Fig. 6c, d.

LiDFOB also has a large anionic radius and relatively small dissociation energy with a high degree of dissociation. Pan et al. [52] prepared an LHCE with LiDFOB, triethyl phosphate (TEP), and 1,1,2,2-tetrafluoroethyl-2,2,3,3 tetrafluoropropyl ether (HFE). This LHCE has high conductivity, low viscosity, and good compatibility with Li anode electrodes. The Li||NMC532 batteries with this LHCE had outstanding capacity retention when charged and discharged at low temperatures. The discharge-specific capacity of Li||NMC532 in LHCE at − 10, − 20, − 30, and − 40 °C is 169, 150, 125, and 90 mAh g^−1^, respectively, which is much higher than its specific discharge capacity in commercial electrolytes. In addition, Fang et al.[53] developed an electrolyte consisting of LiDFOB lithium salt dissolved in 1,2-ethylene sulfite solvent (ES). 1 M LiDFOB–ES electrolyte exhibited a high ionic conductivity of 0.44 mS cm^−1^ at − 40 °C. The Li||LiNi_0.6_Mn_0.2_Co_0.2_O_2_(NMC622) cell with 1 M LiDFOB–ES electrolyte realized a high reversible capacity of 150 mAh g^−1^ and an ultra-high CE of 99.6% at − 30 °C, much higher than that of cells using commercial electrolytes. Nevertheless, LiDFOB is not compatible with DOL solvent with a low freezing point and viscosity. As DOL is prone to polymerization in the presence of water and inorganic lithium salts such as LiDFOB, LiPF_6_, LiBF_4_, etc. resulting in low ionic conductivity. The use of trimethylsilyl isocyanate (Si-NCO) as an electrophilic reagent can remove the water molecules from the DOL/DME mixed solvent and increase the conductivity to 2.16 mS cm^−1^ (− 40 °C), as shown in Fig. 6e [54].

### Weak Li^+^-Solvent Interaction

The de-solvation of Li^+^ is widely considered to be the rate-limiting step at low temperatures [51, 55, 56], thus reducing the de-solvation energy is a key method to improve the low-temperature performance of LMBs. Electrolytes with weak Li^+^-solvent interaction exhibit lower de-solvation energy and a faster de-solvation process. While establishing electrolytes with low de-solvation energy holds great promise, identifying the most effective method poses challenges [57]. The efficacy of these strategies may vary depending on specific application requirements and system configurations.

#### Ether-Based Solvents

**Fig. 7:**
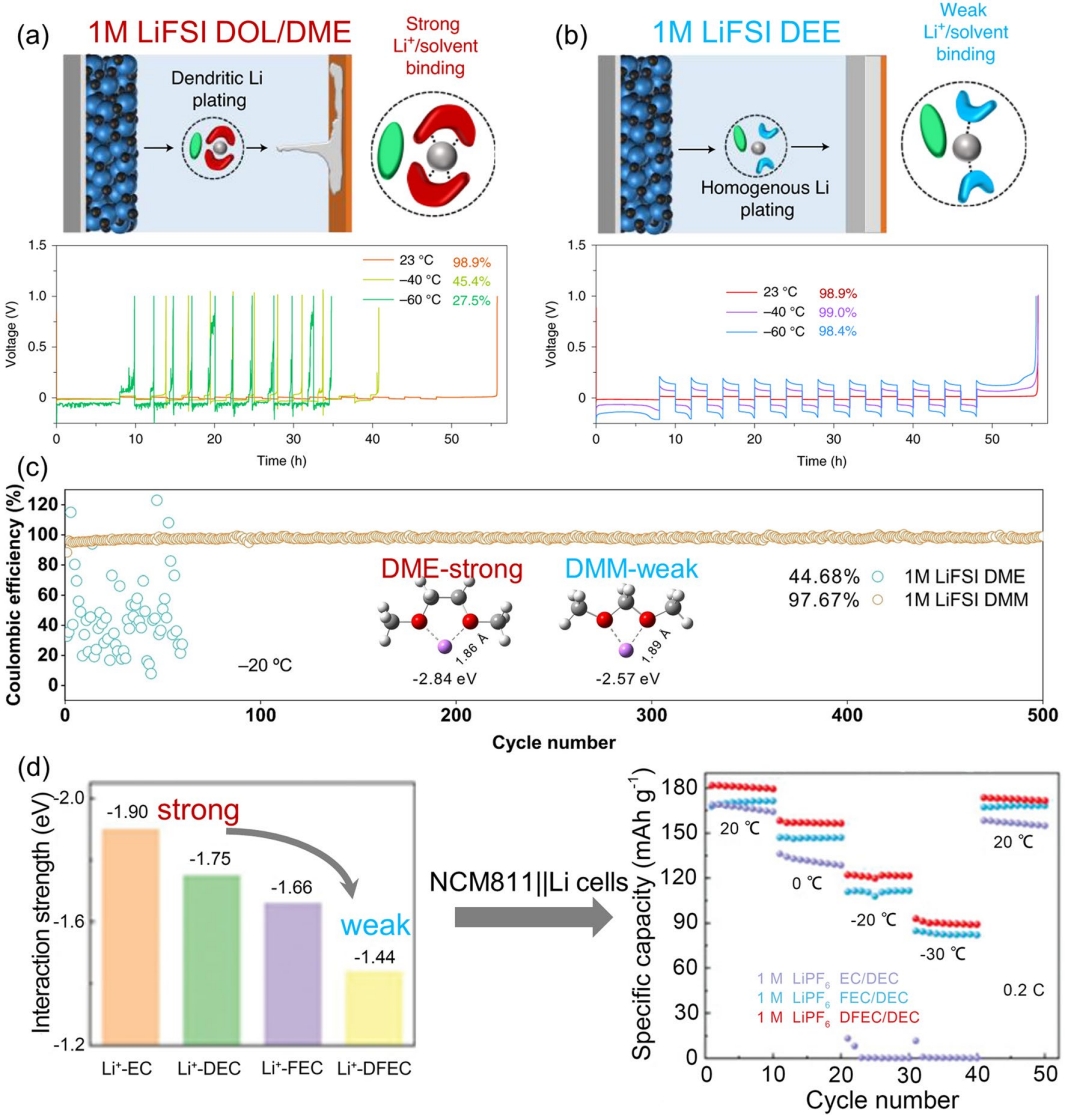
Desolvation mechanisms and corresponding Li||Cu efficiencies for **a** 1 M LiFSI DOL / DME and **b** 1 M LiFSI DEE. Reproduced with permission from Ref. [6]. Copyright 2021, Springer Nature. **c** CE tests of 1 M LiFSI DMM electrolyte at 25 °C,  − 20 °C and − 40 °C, respectively. Reproduced with permission from Ref. [60]. Copyright 2022, Wiley–VCH. **d** The interaction strength between Li^+^ and polar solvent obtained by first-principles calculations and the corresponding performance of NCM811||Li cells at 0.2 C from 20 to − 30 °C. Reproduced with permission from Ref. [21]. Copyright 2021, Wiley–VCH

Liu et al. [6] demonstrated that compared with solvents with strong solvation ability, solvents with weak solvation ability can form a uniform and dense Li deposition (Fig. 7a, b). Dense dendrite-free structures were shown at 23, − 40, and − 60 °C in electrolytes with weakly bonded Li^+^ and diethyl ether (DEE). This structure guaranteed excellent cycle stability of Li||Cu batteries with CEs of 98.9%, 99.0%, and 98.4% at 23, − 40, and − 60 °C, respectively. At − 40 and − 60 °C, 0.1 C rate, the Li||sulfurized polyacrylonitrile (Li||SPAN) full cells using 1 M lithium bis(fluorosulfonyl)imide (LiFSI)/DEE with a SPAN cathode at the loading of 3.5 mAh cm^−2^ exhibited output capacities of 519 and 414 mAh g^−1^, respectively. The same group further reported another electrolyte with weak Li^+^–solvent binding interactions [58]. This electrolyte used 2 M LiFSI in monodentate dibutyl ether (DBE), which is capable of exhibiting excellent electrochemical performance in the temperature range from − 40 to 50 °C. The CEs of Li||Cu cells with LiFSI/DBE at 23, − 40, and 50 °C were 99.0%, 98.2%, and 98.7%, respectively. Li||SPAN full cells using 2 M LiFSI DBE with a SPAN cathode at the loading of 1.2 mAh cm^−2^ display a capacity decay of 0.06% per cycle over 200 cycles. Similar electrolytes of 4 M LiFSI DBE have been reported for Li-SPAN batteries with a high Coulombic efficiency for Li deposition/stripping (~ 99.2%) [59]. The decrease of LiFSI concentration to 2 M allows this electrolyte for low-temperature application but sacrifices the Li metal efficiency from 99.2% to 99.0 at room temperature. As for high-temperature applications, DBE, although not as volatile as DEE, is still volatile, evidenced by the limited cycling performance of 30 cycles.

Ma et al. [60] also reported a weak Li-solvent interaction system of LiFSI/ dimethoxymethane (DMM) and tested the CEs of Li||Cu cells with 1 M LiFSI DMM electrolyte at − 20 °C (98.19%) and − 40 °C (97.87%), as shown in Fig. 7c. The SPAN full cell of this LiFSI DMM system exhibited not only a high first discharge capacity (422.3 mAh g^–1^), but also a favorable capacity retention rate (63.8% after 120 cycles) at − 40 °C. Ding et al. [61] proposed a diethoxymethane (DEM) with ultra-weak solvation ability by shortening the intermediate alkyl chain and increasing the terminal alkyl chain of DME. Li||Cu cells with 1 M LiFSI DEM presented a high CE of 98.7% at − 20 °C. In contrast, the CE of 1 M LiFSI DME is only 68.4% under the same conditions.

Due to the anodic limit, most ether-based electrolytes cannot support high-voltage cathodes. High-concentration electrolytes and fluorinated solvents are promising alternatives that simultaneously enable stable operation under high cutoff voltages and improved Li metal cycling efficiency [62]. By increasing the LiFSI concentration in the carbonate electrolyte to 10 M, Fan et al. [63] formed LiF-rich interface phases on the cathode and anode surfaces. The assembled battery achieved high cycle stability, and the CE of lithium metal plating/stripping is as high as 99.3%. The concept of LHCEs is also promising for reducing de-solvation energy [61]. Compared to HCE, LHCE has a higher concentration of AGG and CIP in its solvation structure. Therefore, using LHCE at low temperatures can effectively reduce desolvation energy. Although the increase in ion pairs leads to a decrease in the conductivity of the electrolyte, it also weakens the coordination between the Li^+^ and the solvent, so the trade-off has to be taken into account to strike a balanced performance. Holoubek et al. [65] paired the LiFSI/DME components with a bis (2,2,2 trifluoro ethyl) ether (BTFE) diluent to prepare 1 M LiFSI BTFE/DME (5:1, 6 M equivalent local concentration). At –40 °C, this electrolyte displayed an ionic conductivity of 0.87 mS cm^–1^, CE of 98.5%, critical current of 0.75 mA cm^–2^, discharge capacity of 109 mAh g^–1^, and capacity retention of 108 mAh g^–1^ (after 100 cycles, 4.3 V cutoff). In addition, in LHCEs, the weakened interaction between Li^+^ and solvents may result in larger Li^+^ transference numbers, which is advantageous for enhancing fast charging of the battery at low temperatures.

#### Fluorinated Solvents

The degree of fluorination greatly influences the de-solvation behavior of Li^+^. With the increase of fluorination degree in EC, FEC, and di-fluoro ethylene carbonate (DFEC) solvents, the interaction between Li^+^ and solvents becomes weaker and weaker. The de-solvation energy of Li^+^ decreases from 1.90 eV in EC to 1.66 eV in FEC and then to 1.44 eV in DFEC, as illustrated in Fig. 7d [21]. At each temperature, electrolytes with weaker Li^+^-solvent binding demonstrate enhanced capacity. Specifically, at − 20 °C, the EC-based electrolyte exhibits stronger Li^+^-solvent binding, resulting in virtually negligible capacity. In contrast, electrolytes based on DFEC and FEC exhibit weaker Li^+^-solvent binding, enabling them to maintain higher capacity even at low temperatures. At − 30 °C, the DFEC and FEC-based electrolytes continue to display superior capacity, reaching 51% and 45% of the room temperature capacity, respectively. LiPF_6_ was used as lithium salt and DEC was used as an additive to configure electrolytes with EC, FEC, and DFEC respectively, and assembled Li||LiNi_0.8_Mn_0.1_Co_0.1_O_2_(NMC811) cells for testing. The capacities of FEC-based electrolytes at − 20 and − 30 °C were 108 and 78 mAh g^–1^ respectively. The capacities of DFEC-based electrolytes at − 20 and − 30 °C were 120 and 93 mAh g^–1^ respectively, all of which were higher than the corresponding values of EC-based electrolytes. This work may greatly inspire regulating the Li-solvent interaction by tuning the fluorination degree.

#### Liquefied Gas-Based Solvents

The liquefied gas electrolytes (LGEs) have polar viscosity and freezing points, making them competitive low-temperature electrolytes for LMBs. Yang et al. [64] published an LGE with an extremely wide operating temperature range (− 78 to 75 °C) that exhibited favorable low-temperature conductivity (4.8 mS cm^−1^, at − 78 °C). They used 1.6 M LiTFSI and 1 M acetonitrile (AN) in fluoromethane (CH_3_F, FM). Because of the weak binding of FM to Li^+^, it led to fast Li^+^ transport and de-solvation. This LGE provided a high CE at cycling conditions of 3 and 3 mAh cm^−2^: 96.4% at − 60 °C and 99.4% at 55 °C.

### SEI with High Li^+^ Mobility

Insufficient kinetics at low temperatures can lead to structural changes in the SEI [66]. If the formed SEI is unstable, the lithium exposed in the electrolyte will continue to trigger side reactions, which will eventually lead to low CE, short cycle life, and the formation of lithium dendrites. The organic SEI layer is lithophilic and increases the de-solvation energy of solvated Li^+^ [66, 67]. In contrast, the inorganic SEI layer is lithiophobic which can support the rapid migration of Li^+^ [68, 69]. Therefore, the SEI with more inorganic components is reasonable because it avoids the destruction of the mechanical strength of the SEI and reduces the volume change [67]. Namely, the inorganic SEI layer can enhance the stability of Li metal anodes [64, 66, 69–71].

**Fig. 8:**
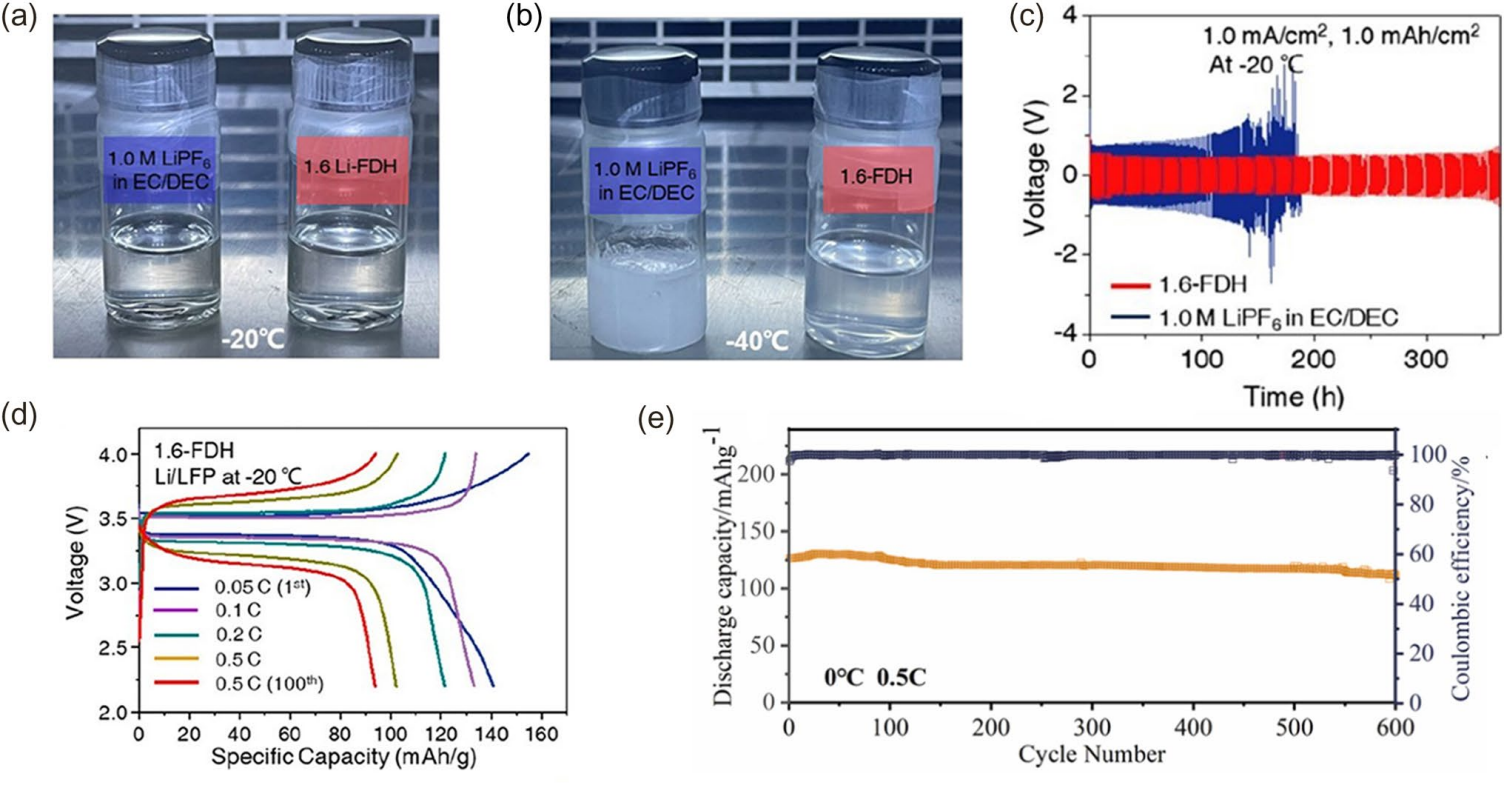
**a** Optical photos of the 1.0 M LiPF_6_ in EC/DEC and 1.6-FDH electrolytes at  − 20 °C. **b** Optical photos of the 1.0 M LiPF_6_ in EC/DEC and 1.6-FDH electrolytes at  − 40 °C. **c** Performance of Li||Li symmetric cells at 1.0 mA cm^−2^ and 1.0 mAh cm^−2^ at –20 °C. **d** Charge/discharge profiles of the Li||LFP cells at the corresponding cycling condition using 1.6-FDH electrolyte at − 20 °C. Reproduced with permission from Ref. [77]. Copyright 2021, American Chemical Society. **e** Long cycling performances at 0.5 C at 0 °C with CPE. Reproduced with permission from Ref. [82]. Copyright 2022, Elsevier

LiFSI possesses not only good solubility and temperature stability but also its anion FSI^−^ can participate in the formation of SEI. Yu et al. [72] dissolved 1.6 M LiFSI in tetrahydrofuran (THF)/2-methyltetrahydrofuran (MTHF) mixed solvent with a volume ratio of 1: 1 and 2 wt% lithium nitrate (LiNO_3_) additive (LiFSI THF/MTHF-LiNO_3_) to prepare electrolyte. Introducing LiNO_3_ as an additive into the electrolyte can lead to the formation of SEI rich in inorganic components, thus improving the cycling performance of the LMB. This electrolyte-derived SEI has favorable kinetics and is capable of reversible Li^+^ plating/stripping at various temperatures. At − 30 °C, this LiFSI THF/MTHF-LiNO_3_ electrolyte provided a high ionic conductivity of 8.1 mS cm^−1^, and the Li||NCA cell with the LiFSI THF/MTHF-LiNO_3_ electrolyte shows 180 mAh g^−1^ after 100 cycles at 0.1 C, which maintained 80% of its room temperature capacity. Moreover, when the temperature returns to 0 °C, the battery is able to recover its initial capacity. It is worth noting that LiNO_3_ has significant applications in lithium-sulfur batteries. When mixed with LPSs (lithium polysulfides), it exhibits synergistic effects that can improve the performance of the LMA. These effects include enhancing the SEI, suppressing lithium dendrite growth, reducing electrode polarization, and inhibiting shuttle effects [73]. A stable SEI was derived from a moderately concentrated electrolyte reported by Wang et al. [74]. This inorganic SEI enriched with Li_2_O and LiF nanocrystals was able to promote the diffusion of Li^+^ and exhibited a wide temperature range (− 40 ~ 60 °C) of applications. Kim et al. [75] proposed an LHCE with an SEI formed with a large number of grain boundaries, which can simultaneously increase the migration rate of Li^+^ in the bulk phase and SEI. 1,1,2,2tetrafluoroethyl-2,2,3,3-tetrafluoropropyl ether (TTE) is a diluent with a low solubility for lithium salts, serving solely as a diluent without participating in the solvation structure of Li^+^. They used TTE to dilute 1.5 M LiFSI/THF and added FEC to it (1.5-TTF), where the ratio of Li^+^ to THF is 2.5. Compared with 5 M LiFSI/THF (5-THF) and 1 M LiPF_6_ EC/DEC (CVE), the transport properties in the SEI of 1.5-TTF are significantly better. The Li||NMC811 full cell retained 75% and 64% of its room temperature capacity at − 20 and − 40 °C, respectively. Shi et al. [76] proposed an amphiphilic diluent, 1,1,2,2-tetrafluoro-3-methoxypropane (TFMP), which simultaneously solvates Li^+^ with its lithiophobic segment and solvates Li^+^ with its lipophilic segment. Unlike TTE, when used as a diluent, TFMP can enter the solvation layer of Li^+^, inducing the self-assembly of a unique core–shell solvation structure in the 1 M LiFSI-TFMP/DME electrolyte. This further decreases DME-Li^+^ coordination, promotes FSI^–^-Li^+^ pairing, significantly enhances ion conductivity, reduces solvation energy, and forms a highly stable and conductive inorganic SEI film. Zheng et al. [77] designed an electrolyte with 1.6 M LiFSI in FEC/DME/HFE (1.6-FDH). The 1.6-FDH electrolyte can remain liquid over a wide range of temperatures. Although at − 20 °C, the commercial 1.0 M LiPF_6_ EC/DEC and 1.6-FDH electrolytes appear to be comparable (Fig. 8a). When the temperature dropped to − 40 °C, the commercial electrolyte was frozen, while the 1.6-FDH electrolyte remained in the liquid state (Fig. 8b). The decomposition of the 1.6-FDH formed an SEI rich in LiF, Li_2_CO_3_, and Li_2_O, which was able to improve the mobility of Li^+^. Li||Li symmetric cell using 1.6-FDH electrolyte achieved a cycle life of greater than 350 h at − 20 °C, compared to about 140 h for commercial electrolytes (Fig. 8c). At − 20 °C, the reversible cycles of Li||LiFePO_4_ (LFP) cells using 1.6-FDH electrolyte exceeded 100 cycles with discharge capacities of 140.4, 127.9, and 111.7 mAh g^−1^ at 0.1, 0.2, and 0.5 C, respectively (Fig. 8d).

A quasi-solid polymer electrolyte forming a dual-layered SEI and amorphous CEI was reported with outstanding low temperature performance [78]. The inner layer is a homogeneous LiF-rich inorganic SEI capable of improving the conductivity of ions. The outer layer is amorphous Li_*x*_BO_*y*_F_*z*_, which can reduce the effect of volume change of the Li metal anode electrode. In addition, the amorphous CEI can stabilize the cathode. With both SEI and CEI, this polymer-based cell performs well at both room temperature and low temperature. The Li||NMC811 provided an initial capacity of ~ 198 mAh g^−1^ at 30 °C, 100 mA g^−1^ and 92 mAh g^−1^ at − 30 °C, 20 mA g^−1^.

In addition to forming inorganic-rich SEI, there are other strategies to improve the mobility of Li^+^ in SEI. Wang et al. [79] constructed a Li_2_O-Ni-based SEI film, an artificial SEI layer with rich grain boundaries, on a three-dimensional Ni-NiO current collector. The low diffusion potential of Li^+^ at the grain boundaries in the SEI layer allowed for rapid migration of Li^+^. The Li||NMC811 full cell with this electrolyte exhibited an area capacity of 3.2 mAh cm^−2^ at − 30 °C. Solid-state electrolytes (SSEs) are nonflammable and can inhibit the formation of lithium dendrites, which are expected to be used in LMBs [80, 81]. Li et al. [82] synthesized a composite polymer electrolyte (CPE) in which the ether compounds formed localized conjugated structural chains to enhance the transfer of Li^+^. Under 0 °C, the LMBs with this CPE showed good cycling performance of over 600 cycles at 0.5 °C, as shown in Fig. 8e. In addition to methods to improve the uniformity of lithium plating, Chen et al. [83] suggested that preventing electron migration to the electrode surface would better enhance the stability of the lithium metal anode, providing another possible solution to improve the stability of LMB at low temperatures.

## Summary and Perspective

This paper summarizes the factors that lead to the poor low-temperature performance of LMBs by analyzing the basic Li^+^ transport steps: (1) low bulk electrolyte conductivity, (2) high de-solvation energy, and (3) sluggish Li^+^ migration in the SEI. To address these limiting steps, emerging strategies in the perspective of electrolyte design are summarized: (i) employing highly dissociated lithium salt to increase the bulk ionic conductivity; (ii) tailoring the solvation structure to minimize the de-solvation energy; (iii) constructing robust SEI that enables fast Li^+^ transport.

Much progress has been made in the study of low-temperature LMBs in recent decades, but there are still several challenges to be explored: (1) To reduce the impedance of charge transfer, it is necessary to reveal the secret of the solvation structure of Li^+^. The influence of the environment (e.g., ion pairs and solvent-anion interactions) surrounding the solvated structure of Li^+^ on the de-solvation process is not well understood. (2) Exploring the relationship between SEI and battery performance is very important. Understanding the role of SEI's organic and inorganic components can guide electrolyte design to form the required SEI. Investigating why LiF-rich SEI has good ion transport properties may be a breakthrough in solving this problem. (3) The pursuit of one aspect tends to ignore the other, for example, electrolytes with low de-solvation energy may have low conductivity. (4) Despite making some progress, the practicality of lithium batteries at low temperatures still faces challenges. In practical applications, there is a need to address a range of considerations, including safety, stability, cost-effectiveness, and scalability. The first two approaches are of utmost importance for future research. The solvation structure of Li^+^ in different electrolyte systems can be investigated through techniques such as molecular dynamics simulations and neutron scattering measurements. These will provide insights into the influence of the surrounding environment, such as ion pairs and solvent-anion interactions, on the de-solvation process of Li^+^. Systematic exploration of the formation mechanism and properties of SEI through techniques like in situ microscopy, electrochemical impedance spectroscopy, and X-ray photoelectron spectroscopy can pave the way for designing an ideal SEI. Search for an optimal solution to obtain a long life, fast charging/discharging and high energy density battery requires systems thinking. Through continuous efforts, the era of rapid development of wide-temperature LMBs is coming.
